# Supplementary material for: Metabolomic Profiling of Adipose Tissue in Type 2 Diabetes: Associations with Obesity and Insulin Resistance
Source: Metabolites. 2024 Jul 26;14(8):411. doi: 10.3390/metabo14080411 (PMC11356712; doi:10.3390/metabo14080411)
Supplement: Supplementary file 1 [file metabolites-14-00411-s001.zip › Supplementary Figures.pdf]

Supplementary Figures

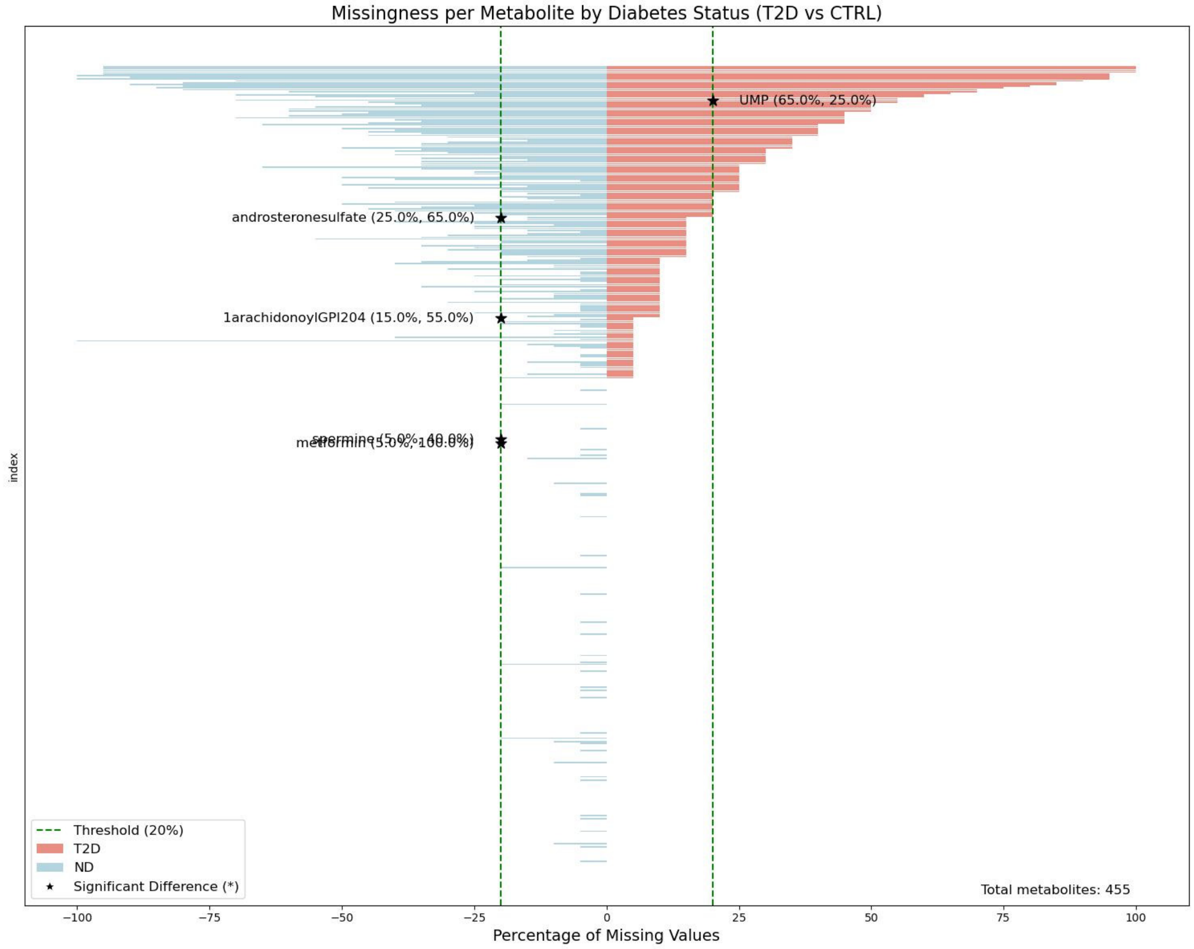

Supplementary Figure S1. Missingness of metabolites in by diabetes status (T2D vs. Control). This figure illustrates the percentage of missing values for all identified metabolites in adipose tissue samples from subjects with and without T2D. Metabolites missing in more than 20% of the samples in both the T2D and ND groups (indicated by the dashed green line) were excluded from further analyses.

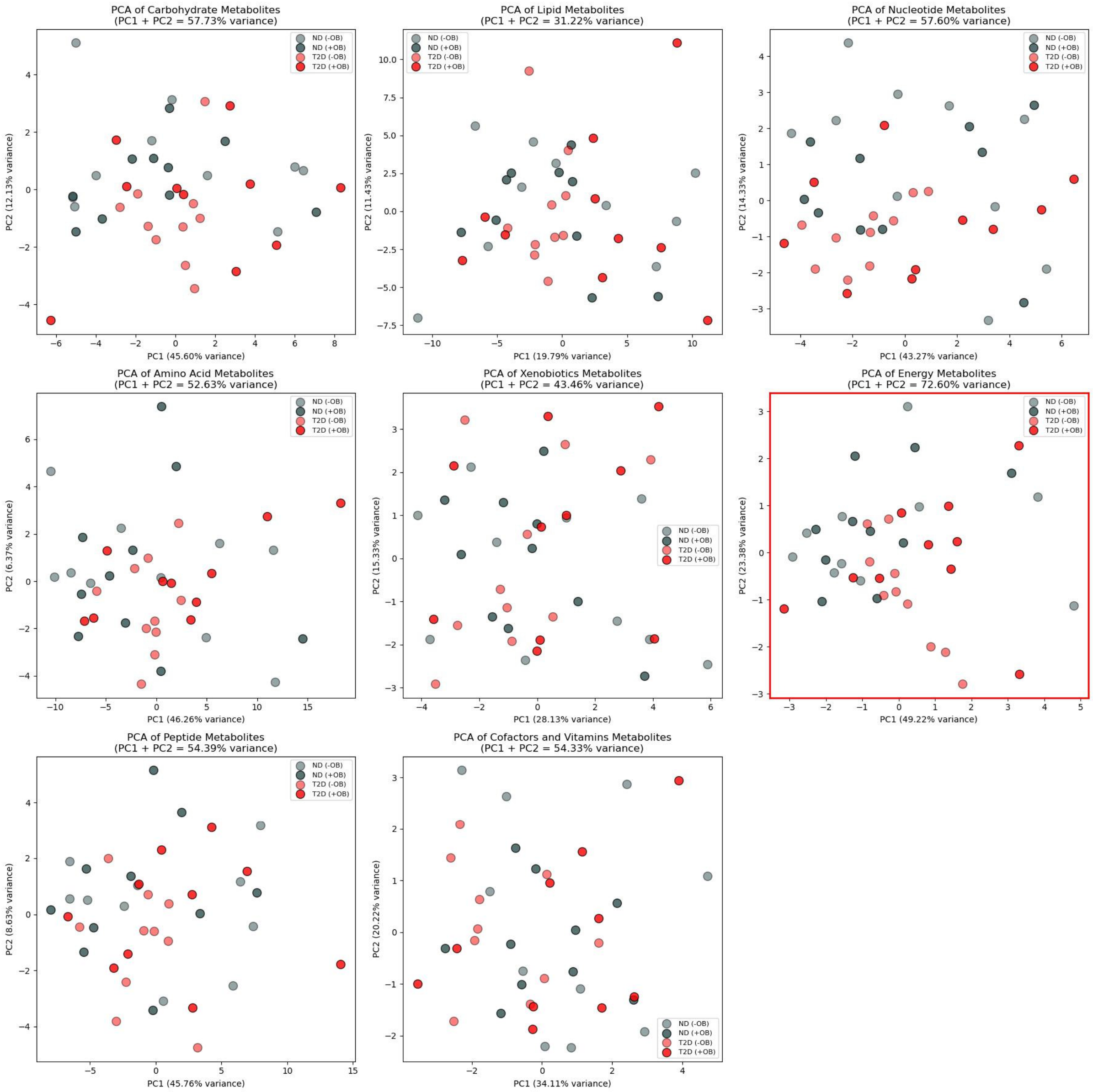

Supplementary Figure S2. Principal Component Analysis (PCA) of metabolite type categories. In this multi-panel image, each panel represents the PCA results of a metabolite class across the four distinct groups formed by Type 2 diabetes (T2D) and obesity (OB) conditions. The groups are: i) ND (-OB): subjects without T2D and without obesity, represented with a light grey circle ii) ND (+OB): subjects without T2D and with obesity, with the darker grey circles iii) T2D (-OB): subjects with T2D and without obesity illustrated with pink circles and iv) T2D (+OB): subject both T2D and obesity, shown as red circles. Principal components 1 and 2, explaining the most variance percentage, are shown in axes x and y respectively.

The clustering of points indicates similarity in metabolite profiles among the samples. Annotated with a red border, the PCA of the metabolites within the Energy category, show a separation of the subjects with T2D without obesity from the other groups that reflects the differences in their metabolite profiles.
